# Reliability of EuroSCORE II on Prediction of Thirty-Day Mortality and Long-Term Results in Patients Treated with Sutureless Valves

**DOI:** 10.3390/jcm13133986

**Published:** 2024-07-08

**Authors:** Lorenzo Di Bacco, Michele D’Alonzo, Massimo Baudo, Andrea Montisci, Marco Di Eusanio, Thierry Folliguet, Marco Solinas, Antonio Miceli, Theodor Fischlein, Fabrizio Rosati, Claudio Muneretto

**Affiliations:** 1 Unit of Cardiac Surgery, Univesity of Brescia, 25123 Brescia, Italy; lorenzo.dibacco@hotmail.it (L.D.B.); montisci.andrea@yahoo.it (A.M.); rosati.fabri@gmail.com (F.R.); claudio.muneretto@unibs.it (C.M.); 2 Cardiothoracic Surgery, Lankenau Institute for Medical Research, Wynnewood, PA 19096, USA; massimo.baudo@icloud.com; 3 Cardiac Surgery Unit, Lancisi Cardiovascular Center, Polytechnic University of Marche, 60121 Ancona, Italy; marco.dieusanio@ospedaliriuniti.marche.it; 4 Unit of Cardiac Surgery, Henri Mondor Hospital, 94010 Creteil, France; thierry.folliguet@aphp.fr; 5 Monasterio Foundation Heart Hospital, 54100 Massa, Italy; solinas@ftgm.it; 6 Department of Minimally Invasive Cardiac Surgery, Sant’Ambrogio Hospital, 20122 Milan, Italy; antoniomiceli79@alice.it; 7 Department of Cardiac Surgery, Paracelsus Medical University, 90419 Nuremberg, Germany; t.fischlein@klinikum-nuernberg.de

**Keywords:** EuroSCORE II, sutureless aortic valves, ROC curve, minimally invasive cardiac surgery

## Abstract

**Background:** EuroSCORE II (ES2) is a reliable tool for preoperative cardiac surgery mortality risk prediction; however, a patient’s age, a surgical procedure’s weight and the new devices available may cause its accuracy to drift. We sought to investigate ES2 performance related to the surgical risk and late mortality estimation in patients who underwent aortic valve replacement (AVR) with sutureless valves. **Methods**: Between 2012 and 2021, a total of 1126 patients with isolated aortic stenosis who underwent surgical AVR by means of sutureless valves were retrospectively collected from six European centers. Patients were stratified into three groups according to the EuroSCORE II risk classes (ES2 < 4%, ES2 4–8% and ES2 > 8%). The accuracy of ES2 in estimating mortality risk was assessed using the standardized mortality ratio (O/E ratio), ROC curves (AUC) and Hosmer–Lemeshow (HL) test for goodness-of-fit. **Results**: The overall observed mortality was 3.0% (predicted mortality ES2: 5.39%) with an observed/expected (O/E) ratio of 0.64 (confidential interval (CI): 0.49–0.89). In our population, ES2 showed a moderate discriminating power (AUC 0.65, 95%CI 0.56–0.72, *p* < 0.001; HL *p* = 0.798). Good accuracy was found in patients with ES2 < 4% (O/E ratio 0.54, 95%CI 0.23–1.20, AUC 0.75, *p* < 0.001, HL *p* = 0.999) and for patients with an age < 75 years (O/E ratio 0.98, 95%CI 0.45–1.96, AUC 0.76, *p* = 0.004, HL *p* = 0.762). Moderate discrimination was observed for ES2 in the estimation of long-term risk of mortality (AUC 0.64, 95%CI: 0.60–0.68, *p* < 0.001). **Conclusions**: EuroSCORE II showed good accuracy in patients with an age < 75 years and patients with ES2 < 4%, while overestimating risk in the other subgroups. A recalibration of the model should be taken into account based on the complexity of actual patients and impact of new technologies.

## 1. Introduction

Results of transcatheter aortic valve implantation (TAVI) have dramatically changed the treatment of degenerative aortic stenosis [1,2,3]. Consequently, a careful preoperative risk assessment is now required to establish the best treatment option between surgery and TAVI [4]. In this setting, surgical risk scores are useful tools for preoperative risk prediction and decision-making processes [5,6]. EuroSCORE II (ES2) is an easy, user-friendly risk score for operative mortality prediction [7,8]; however, improvement in clinical practice, the spread of minimally invasive surgical approaches and the increasing burden of patients’ comorbidities [9,10,11] may cause its predictive accuracy to drift. In particular, patients’ ages have been demonstrated to potentially jeopardize the reliability and the predictive power of ES2 [12]: its calibration decreases in patients over 70 years at intermediate to high surgical risk [13], and it tends to overestimate risk in octogenarians [14].

The introduction in the surgical armamentarium of sutureless aortic valves (Su) for patients undergoing aortic valve replacement (AVR) reduced operative risks and times [15,16] with excellent safety profiles observed even in elderly patients with intermediate and high surgical risk [17,18]. However, the use of such new devices may represent an additional confounding factor in the use of ES2 to predict preoperative risk. As a matter of fact, surgical risk scores do not take into account the usage of new technologies, such Su-AVR and TAVI, for the advantages and potential onset of device complications such as paravalvular leaks (PVL) and permanent pacemaker implantation, well known as independent predictors for long-term survival.

In this study, we sought to investigate the performance of EuroSCORE II in predicting early and long-term mortality in different surgical risk classes using a larger European dataset in patients who underwent Su-AVR.

## 2. Materials and Methods

Data from a total of 1126 consecutive patients who had undergone isolated Su-AVR by means of sutureless Perceval S valve (CorCym Srl, Saluggia, Italy) were retrospectively collected between January 2012 and December 2021 from six European centers. Patients who had undergone combined procedures were excluded from this study.

Preoperative data were retrieved from the institutional databases of each center, and the entire population was stratified into three groups according to the EuroSCORE II surgical risk categories [low-risk (L-ES2): EuroSCORE II < 4; intermediate-risk (I-ES2): 4 ≤ EuroSCORE II < 8; high-risk (H-ES2): ≥8].

Patients were judged unsuitable for Perceval implantation if the ratio between the sinotubular junction (STJ) and aortic annulus diameter was over 1.3. Measures of STJ, annulus and distance between the annulus and STJ were obtained with ECG-gated angio-CT scans or transthoracic echocardiography.

Sievers type I bicuspid aortic valve was considered a relative contraindication, while patients with bicuspid valve Sievers type 0 (true bicuspid without raphe) were excluded.

The primary outcomes were 30-day mortality and EuroSCORE II calibration for mortality prediction. Secondary outcomes were long-term mortality, major adverse cardiac and cerebrovascular events at follow-up, EuroSCORE II performance for mortality risk prediction at follow-up and identification of independent predictors for mortality at follow-up.

### Statistical Analysis

The distribution normality of continuous variables was tested with the Kolmogorov–Smirnov test. Data conforming to normal distribution were described as mean with standard deviation, while non-normal distribution was described as median with interquartile range. ANOVA and Kruskal–Wallis tests were used for normal and non-normal intergroup comparisons, respectively. Categorical variables were expressed as frequency and percentage and compared with the Pearson Chi-square test or Fisher’s exact test when the expected frequencies of one or more cells was less than 5.

Standardized mortality ratio (observed/expected ratio, O/E ratio) s was calculated at 30 days with a 95% confidence interval (CI).

Kaplan–Meier curves of the three groups were charted for late overall mortality, cardiac-related mortality and MACCEs with 95%CI, and compared using a log-rank test. Univariable and multivariable Cox regression analyses were used to investigate the effect of preoperative characteristics on survival.

The discriminatory capacity of the EuroSCORE II on 30-day mortality was evaluated by the area under the receiver operating characteristics (ROC) curve (AUC). Calibration refers to the agreement between observed and predicted in-hospital mortality. Overall model calibration was assessed by comparing observed and predicted mortality in 10 equally sized subgroups in increasing order of patient risk, according to the Hosmer–Lemeshow (HL) test for goodness-of-fit. An HL *p*-value > 0.05 indicates a well-calibrated model for the study population.

The statistical analyses were performed with SPSS version 26.0 (SPSS Inc., Chicago, IL, USA). The values were statistically significant at *p* ≤ 0.05.

## 3. Results

Of the 1126 patients included in this study, 406 (36.0%) were categorized as L-ES2, 545 (48.4%) as I-ES2 and 175 (15.6%) as H-ES2. Baseline characteristics are described in Supplemental Table S1. The median age of the population was 79 years (interquartile range [IQR] 25th–75th: 73–83 years), 36.4% were male, and the median EuroSCORE II was 5.39% (IQR 25th–75th: 3.19–7.30%).

Significant differences were reported in patients’ demographics between the three risk groups. Specifically, age, gender, body surface area (BSA), dyslipidemia, diabetes, previous atrial fibrillation, peripheral artery disease, previous CABG, previous stroke, GFR < 30 mL/min, low ejection fraction, NYHA III/IV, mitral regurgitation ≥ II, COPD and echocardiographic data were significantly different between groups, thus reflecting the increase in patients’ complexity according to the increase in risk profile (Table S1).

A minimally invasive approach was used in 792 patients (70.3%): 644 patients had a partial upper “J”-shaped hemisternotomy, while 148 patients received a right anterior minithoracotomy. The remaining cases were performed through median sternotomy. Non-elective surgery was more frequent in the H-ES2 group (L-ES2: 0.49%, I-ES2: 2.02%, H-ES2: 2.86%, *p* = 0.019).

### 3.1. 30-Day Mortality

**Figure 1:**
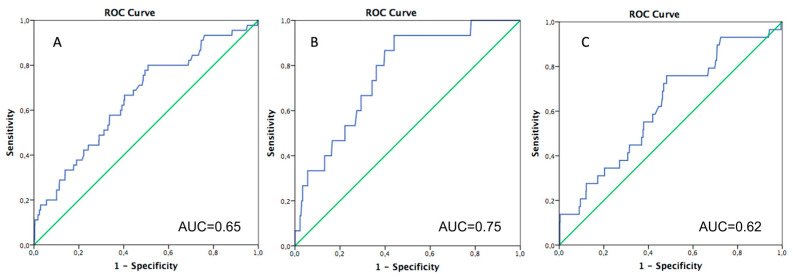
(**A**) ROC for EuroSCORE II in the overall population. (**B**) ROC for EuroSCORE II in patients younger than 75 years old. (**C**) ROC for EuroSCORE II in patients > 75 years.

Overall, 30-day mortality was 3.0%, and no differences were reported between the three groups (LES2: 2.2% vs. IES2: 3.5% vs. HES2: 3.4%, *p* = 0.568) (Table 1). The standardized mortality rate (SMR) showed that EuroSCORE II slightly overestimated the risk of mortality in the overall population with an observed/expected (O/E) ratio of 0.64 [95%CI 49–0.89] (Table 2A). Receiver operating characteristic (ROC) curves of the EuroSCORE II for 30-day mortality showed an area under the curve (AUC) of 0.65, 95%CI: 0.56–0.72, *p* < 0.001 (Figure 1A), with an acceptable predictive power of the score. In addition, ROC statistics revealed a EuroSCORE II score of 4.7% as the optimal cutoff point for predictions of mortality (sensitivity 0.80, 1-specificity 0.51).

**Figure 2:**
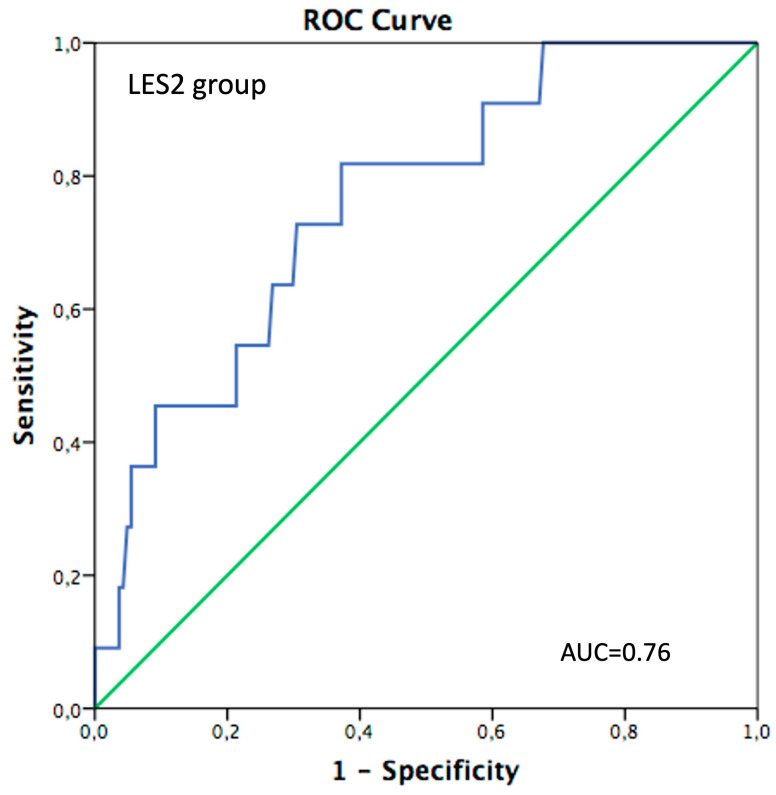
ROC for EuroSCORE II in patients with low surgical risk (ES < 4%).

The EuroSCORE II was adequately calibrated in the L-ES2 group with an O/E ratio of 0.98 (95%CI 0.45–1.96, AUC: 0.76) (Figure 2), while overestimation was observed in both intermediate (O/E ratio: 0.61, 95%CI: 0.37–0.96, AUC: 0.64) and high surgical risk (O/E ratio: 0.3, 95%CI: 0.11–0.65, AUC: 0.58) groups (Table 2A). Age-stratified ROC curves of the EuroSCORE II for 30-day mortality showed an AUC of 0.75, 95%CI: 0.65–0.87, (*p* = 0.001) with an observed/expected ratio of 0.54 (95%CI 0.23–1.20) in patients with an age < 75 years, while the AUC decreased in patients with an age ≥ 75 years (AUC 0.62, 95%CI: 0.50–0.71, *p* = 0.027) with an E/O ratio of 0.47 (95%CI 0.22–0.71), thus showing younger patients having an optimal predictive power over the score (Table 2B) (Figure 1B,C).

**Figure 3:**
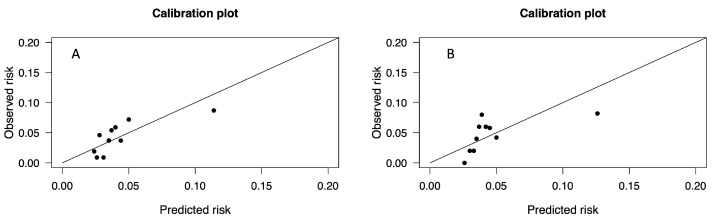
(**A**) Calibration plot for EuroSCORE II in the overall population. (**B**) Calibration plot in patients younger than 75 years.

Hosmer–Lemeshow goodness-of-fit test revealed an acceptable calibration in the overall population and in patients younger than 75 years old (*p* = 0.399 and *p* = 0.534, respectively) (Figure 3A,B) (Table 2B).

Binary logistic regression showed that age over 75 years did not increase the risk of 30-day mortality (odds ratio 0.66, 95%CI 0.32–1.34, *p* = 0.256), while mitral valve regurgitation of a grade ≥ II was identified as a predictor of mortality (OR 2.56, 95% 1.04–5.72, *p* = 0.040).

### 3.2. Follow-Up

**Figure 4:**
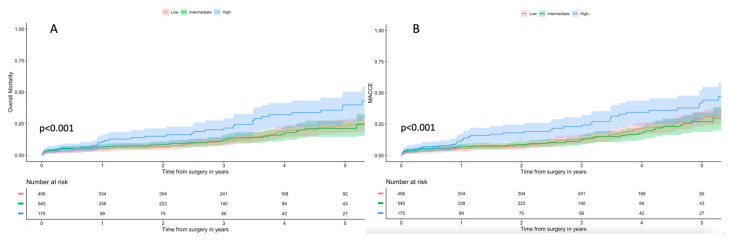
(**A**) Kaplan–Meier curves for overall survival in patients at low, intermediate and high surgical risk. (**B**) Kaplan–Meier curves for survival freedom from MACCE in patients at low, intermediate and high surgical risk.

The mean follow-up time was 26 months (25–75 IQR: 9–49 months). All-cause death was significantly higher in the H-ES2 group during follow-up (36 months: L-ES2: 11.2%, 95% IC: 7.9–14.5% vs. I-ES2: 11.6%, 95% IC: 8.1–15.1% vs. H-ES: 21.6%, 95% IC: 13.9–29.3%; 60 months: L-ES2: 24.0%, 95% IC: 18.7–29.3% vs. I-ES2: 21.3%, 95% IC: 15.2–27.4% vs. H-ES2: 39.9%, 95% IC: 29.4–50.5%, *p* < 0.001) (Figure 4A). Moreover, cardiac-related death was higher in the H-ES2 group at 60 months, while no differences were reported between the L-ES2 and I-ES2 groups (36 months: L-ES2: 6.3%, 95% IC: 3.7–8.9% vs. I-ES2: 7.0%, 95% IC: 3.7–10.3% vs. H-ES: 9.7%, 95% IC: 4.0–15.4%; 60 months: L-ES2: 11.1%, 95% IC: 6.8–15.4% vs. I-ES2: 16.2%, 95% IC: 7.2–25.2% vs. H-ES2: 23.4%, 95% IC: 12.8–34.0%, *p* = 0.017).

Kaplan–Meier curves for cumulative incidence of composite adverse events (MACCE: all-cause death, myocardial infarction, aortic regurgitation, bleeding, endocarditis, reoperation) during follow-up depicted a significantly higher incidence of events in the H-ES2 group (60 months: L-ES2: 30.0%, 95% IC: 24.2–35.8% vs. I-ES2: 29.9%, 95% IC: 23.8–36.0% vs. H-ES2: 44.3%, 95% IC: 33.8–54.8%, *p* < 0.001) (Figure 4B).

The results of the Cox regression analysis for all-cause death are presented in Table 3. At univariate analysis EuroSCORE II > 8%, male gender, preoperative atrial fibrillation and advanced NYHA class (III–IV) were identified as independent predictors of all-cause death. After multivariable analysis adjustment, only EuroSCORE > 8% (HR: 1.82, 95%CI: 1.29–2.56, *p* < 0.001) and preoperative atrial fibrillation (HR: 1.74, 95%CI: 1.14–2.65, *p* < 0.009) were identified as independent predictors of all-cause death.

**Figure 5:**
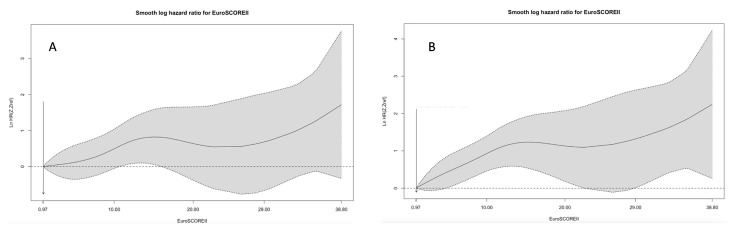
(**A**) Continuous relationship between EuroSCORE II and HR for mortality based on restricted cubic spline models (univariable cox regression). (**B**) Continuous relationship between EuroSCORE II and HR for mortality based on restricted cubic spline models (multivariable Cox regression).

Figure 5 shows a continuous relationship between EuroSCORE II and HR for mortality based on restricted cubic spline models. Receiver operating characteristic curves of the EuroSCORE II for mortality at follow-up showed an AUC of 0.64, 95%CI: 0.60–0.68, *p* < 0.001. The ROC statistics revealed a EuroSCORE II score of 6.9% as the optimal cutoff point for the prediction of mortality (sensitivity 0.62, 1-specificity 0.36).

## 4. Discussion

This multi-institutional study represented a “real-world” experience evaluating the performance of EuroSCORE II in predicting 30-day and long-term mortality on a large population of patients undergoing Su-AVR.

The major findings of our study are as follows:

1. EuroSCORE II overestimated the risk of mortality with an E/O ratio of 0.64 (95% IC: 0.49–0.89) in patients who underwent Su-AVR;
2. The ROC curve tested on the whole population showed an acceptable accuracy of EuroSCORE II in estimating 30-day mortality with an AUC of 0.65;
3. In the L-ES2 subgroup, EuroSCORE II had good predictive power with an E/O ratio of 0.98 (0.45–1.96);
4. In patients younger than 75 years, the ROC curve showed good estimation performance in 30-day mortality with an AUC of 0.75. Suboptimal results were observed in patients older than 75 years, with an AUC of 0.61;
5. The ROC curve showed a performance barely acceptable when ES II was used to estimate the risk of mortality at 60 months follow-up (AUC 0.64).

The performance of EuroSCORE II has been extensively evaluated in several studies reporting an improvement in risk assessment when compared to the previous version of Logistic and Addictive EuroSCORE I [7,8]. Nevertheless, these validation studies were mostly performed on relatively young patients and do not reflect the current landscape [5].

Increased life expectancy has led to a progressive increase in the median age of patients requiring a cardiac intervention. Conversely, the increase in patients’ complexity did not lead to a significant increase in operative mortality, mainly related to an improvement in surgical, anesthesiologic and perioperative care [9,10]. As a result, risk stratification by means of proper scores is considered mandatory in order to establish the best therapeutic strategy, especially in elderly patients with aortic stenosis. The choice between a transcatheter or surgical approach strictly depends on the preoperative risk assessment [19,20].

It has been shown that age, comorbidities and the weight of the planned surgical procedure might influence the discriminating capability of such risk scores. In this setting, aortic valve replacement procedures have been reported to reduce the discrimination of EuroSCORE II [9,10,11,12] when weighted with other surgical procedures.

Results of the present study showed the observed mortality in the whole population to be lower than the expected mortality calculated with EuroSCORE II (observed 3.02%/expected 5.39%), thus leading to a significant risk of overestimation in the intermediate- (O/E ratio: 0.61, 95%CI: 0.37–0.96) and high-risk (O/E ratio: 0.3, 95%CI: 0.11–0.65) groups. Onorati et al. already reported this “overestimation” tendency in high-risk patients with an observed mortality rate in intermediate (observed 3.0% vs. expected 5.8%)- and high (observed 2.1% vs. expected 15.4%)-surgical-risk patients lower than expected [21]. Similarly, Howell and colleagues investigated the low discrimination power of the ES II to predict mortality in high-risk patients reporting an AUC of 0.67 and a failure in the calibration test [10]. A similar attitude was observed in TAVI patients, where EuroSCORE II overestimated the observed mortality, thus reinforcing the concept that minimally invasive procedures may influence the incidence of mortality, suggesting that the effect on the predictive power of the weight of the intervention should be tested [22,23].

Several studies demonstrated that elderly patients represent a special population in cardiac surgery. Provenchère et al. found low calibration of EuroSCORE II in octogenarian patients undergoing cardiac surgery [14], and Poullis et al. reported an AUC below 0.7 in patients > 70 years old receiving AVR [13]. Consistent with these previous results, the present study reported good predictive power in patients younger than 75 years old (AUC 0.75, *p* < 0.001), while the AUC decreased in elderly patients with an overestimation of risk [13,14].

Nevertheless, in the subgroup of younger patients (age < 75 years), we reported fair calibration when assessed by means of the Hosmer–Lemeshow goodness-of-fit test, similarly to previous results obtained merely with EuroSCORE II [7,8], thus suggesting age has a relevant impact on the performance of the risk calculator. This finding could be explained with the relative under-representation of elderly patients in the validation dataset of EuroSCORE II [5]. Since patients’ age represents a discriminant in the decision-making process of treatment of severe aortic stenosis and the latest VHD guidelines have recommended TAVI in patients over 75 years, our results suggest a word of caution. Moreover, EuroSCORE II does not take into account some anatomical (aortic calcification, aortic annulus dimensions) and biological (frailty, organ function) characteristics that, if included, could increase its accuracy. A recalibration or update of EuroSCORE II should be seriously considered. Nowadays, the best way to guide clinical practice is The Heart Team approach [24]. In this field, machine learning has demonstrated itself to be a valid option for improving decision-making processes in the evaluation of preoperative risk for aortic valve replacement [25].

According to our findings, the performance of a surgical risk score should not only be limited to short-term/30-day mortality but also consider the capability to properly estimate mid- and long-term outcomes [24]. In this regard, high-risk patients may take advantage of cardiac surgery in the short term; however, they might be affected by poorer mid- and long-term outcomes related to their comorbidities [26]. Hence, risk stratification on long-term outcomes is of paramount importance beyond immediate results. Predictive factors influencing 30-day mortality may not play a role in determining outcomes mid- and long-term, whereas other neglected predictors might be determinant. We demonstrated that EuroSCORE II has a low accuracy for the estimation of mortality at mid- and long-term follow-up. Indeed, according to Barili et al., our results pointed out a low calibration of EuroSCORE II in predicting 5-year mortality [27].

A similar phenomenon was reported for the STS score by Ishimizu and colleagues, who analyzed the 5-year outcomes of 2588 patients who underwent TAVI. This study showed that patients at high surgical risk had a higher mortality when compared to intermediate- and low-risk patients, and high surgical risk was found to be an independent predictor of mortality. Despite these findings, the calibration of STS score on the prediction of long-term-results remains poor (AUC 0.63) [23].

The main limitation of this study is its retrospective nature, and the results may have been affected by selection bias and unknown potential confounders. Moreover, the analysis in the present study focused only on the performance of EuroSCORE II.

## 5. Conclusions

Considering our results, we suggest a recalibration of EuroSCORE II in determining the risk of elderly patients, keeping in mind that the decision on surgical versus percutaneous interventions should be drawn based on an updated tool. The incoming improvement in the EuroSCORE II should also consider other contemporary outcome predictors, such as frailty and anatomical patients’ features. Nowadays, machine learning should be taken into account as an integrative tool in improving decision-making processes choosing the best therapeutic strategy for every patient.

## Figures and Tables

**Table 1:** Operative and early outcomes.

Variables	SUAVR (n = 1126 pts)		Low Risk (n = 406 pts)		Intermediate Risk (n = 545)		High Risk (n = 175)		*p*-Value
	n (%)		n (%)		n (%)		n (%)		
Surgical approach									
Sternotomy	284	25.2%	95	23.4%	147	27.0%	57	32.6%	<0.001
Ministernotomy	674	59.9%	297	73.2%	259	47.5%	118	67.4%	
Right anterior thoracotomy	168	14.9%	14	3.4%	139	25.5%	0	0.0%	
Non-elective procedure	18	1.6%	2	0.5%	11	2.0%	5	2.9%	0.019
Aortic cross-clamp (min) (mean ± SD)	43.9	21.2	41.4	18.8	48.7	22.7	36.4	19.6	<0.001
CPB (min) (mean ± SD)	69.4	31.5	63.8	28.4	77.5	32.5	61.6	31.8	<0.001
Intraprocedural mortality	7	0.6%	3	0.7%	2	0.4%	2	1.1%	0.568
30-Day mortality	34	3.0%	9	2.2%	19	3.5%	6	3.4%	0.540
AKI	37	3.3%	16	3.9%	12	2.2%	9	5.1%	0.048
Stroke/TIA	38	3.4%	12	2.9%	18	3.3%	8	4.6%	0.021
Life-threatening bleeding	59	5.2%	20	4.9%	30	5.5%	9	5.1%	0.985
Intraprocedural AMI	1	0.1%	0	0.0%	0	0.0%	1	0.6%	0.987
Permanent PM implantation	79	7.0%	30	7.4%	35	6.4%	14	8.0%	0.669
Paravalvular leak ≥ 2	11	1.0%	3	0.7%	6	1.1%	2	1.1%	0.845
Endocarditis	19	1.7%	2	0.5%	12	2.2%	5	2.8%	0.063
Pneumonia	35	3.1%	5	1.2%	23	4.2%	7	4.0%	0.125
Transfusions	343	30.5%	93	22.9%	179	32.8%	71	40.6%	<0.001
DSWI	11	1.0%	3	0.7%	6	1.1%	2	1.1%	0.816

CPB: Cardiopulmonary Bypass, AKI: Acute Kidney Failure, PM: Pacemaker, AMI: Acute Myocardial Infarction, DSWI: Deep Sternal Wound Infection.

**Table 2:** A. EuroSCORE II calibration in the overall population and patients younger and older than 75 years old. B. EuroSCORE II calibration according to different preoperative risk categories (LES2, IES2, HES2).

**A**	**Low Risk**	**Intermediate Risk**	**High Risk**
	**n = 406**	**n = 545**	**n = 175**
O/E ratio (95%CI)	0.98 (0.45–1.96)	0.61 (0.37–0.96)	0.30 (0.11–0.65)
AUROC (95%CI)	0.76 (0.64–0.89)	0.64 (0.53–0.74)	0.58 (0.41–0.75)
AUROC *p*-value	*p* = 0.004	*p* = 0.023	*p* = 0.422
Bonferroni correction	0.008	0.046	0.844
HL goodness-of-fit test			
calibration p-value	0.381	0.061	0.098
Bonferroni correction	0.762	0.122	0.196
**B**	**Overall Population**	**Age ≤ 75 yrs**	**Age > 75**
	**n = 1126**	**n = 292**	**n = 834**
O/E ratio (95%CI)	0.64 (0.49–0.89)	0.54 (0.23–1.20)	0.47 (0.22–0.71)
AUROC (95%CI)	0.65 (0.56–0.72)	0.75 (0.65–0.87)	0.62 (0.50–0.71)
AUROC p-value	*p* < 0.001	*p* < 0.001	*p* = 0.027
Bonferroni correction	<0.001	<0.001	0.844
HL goodness-of-fit test			
Calibration p-value	0.399	0.534	0.046
Bonferroni Correction	0.798	0.999	0.092

O/E: Observed/Expected; AUROC: Area Under Receiver Operating Curve; HL: Hosmer–Lemeshow.

**Table 3:** Univariable and multivariable Cox regression for long-term mortality.

	Univariable				Multivariable			
	HR	LCI	UCI	*p*-Value	HR	LCI	UCI	*p*-Value
ESII ≥ 8% (versus low and intermediate risk)	2.10	1.51	2.92	<0.001	1.82	1.29	2.56	<0.001
Age	1.03	1.00	1.05	0.029				
Male Gender	1.38	1.04	1.83	0.025				
BSA	0.61	0.28	1.30	0.203				
Active Smoker	0.85	0.55	1.29	0.456				
Diabetes Mellitus	1.31	0.97	1.77	0.068				
Previous Atrial Fibrillation	2.40	1.62	3.57	<0.001	1.74	1.14	2.65	0.009
PAD	1.06	0.74	1.51	0.729				
Previous CABG	0.98	0.40	2.41	0.974				
Previous Stroke/TIA	1.57	0.98	2.52	0.055				
COPD	1.19	0.84	1.71	0.318				
Advanced NYHA	1.36	1.01	1.84	0.039				
CKF	1.59	0.90	1.78	0.109				
LVEF < 30%	0.98	0.97	0.99	0.008				
Bicuspid Aortic Valve	0.36	0.16	0.83	0.016				
Preoperative MR > Grade II	1.06	0.63	1.78	0.802				

ESII: EuroSCORE II; BSA: Body Mass Index; PAD: Peripheral Artery Disease; CABG: Coronary Artery Bypass Grafting; TIA: Transitory Ischemic Attack; COPD: Chronic Obstructive Pulmonary Disease; CKF: Chronic Kidney Failure; LVEF: Left Ventricular Ejection Fraction; MR: Mitral Regurgitation.

## Data Availability

Dataset available on reasonable request from the authors.

## Supplementary Materials

The following supporting information can be downloaded at: https://www.mdpi.com/article/10.3390/jcm13133986/s1, Table S1: Preoperative patients’ characteristics.
