# Supplementary material for: Reliability of EuroSCORE II on Prediction of Thirty-Day Mortality and Long-Term Results in Patients Treated with Sutureless Valves
Source: J Clin Med. 2024 Jul 8;13(13):3986. doi: 10.3390/jcm13133986 (PMC11242606; doi:10.3390/jcm13133986)
Supplement: Supplementary file 1 [file jcm-13-03986-s001.zip › jcm-3027437-supplementary.pdf]

Table S1. Preoperative patients' characteristics.

Variable	SUAVR (n=1126 pts)		Low risk (n=406 pts)		Intermediate risk (n=545)		High-risk (n=175)		p-value
	n (%)		n (%)		n (%)		n (%)		
Age (years) (median, 25th-75th iQR)	79	(73-83)	78	(75-82)	79	(75-83)	80	(77-84)	<0.001
Male Gender	410	36.4%	171	42.1%	183	33.6%	56	32.0%	0.016
BMI (Kg/m2)	27.1	(4.6%)	27. 4	4.5	27.1	4.9	26.5	4.1	0.115
BSA (m2) (median, 25th-75th iQR)	1.8	(1.6 - 1.9)	1.8	(1.7 - 1.9)	1.8	(1.6-1.9)	1.8	(1.6-1.9)	0.048
Current smoker	349	30.1%	60	14.8%	218	40.0%	71	40.6%	<0.001
Hypertension	943	83.7%	346	85.2%	449	82.4%	148	84.6%	0.447
Dyslipidemia	636	56.5%	188	46.3%	304	55.8%	144	82.3%	<0.001
Diabetes mellitus	362	32.1%	93	22.9%	201	36.9%	68	38.8%	<0.001
Atrial fibrillation	127	11.3%	28	6.9%	55	10.1%	44	25.1%	0.001
Peripheral artery disease	225	20.0%	53	13.0%	124	22.8%	48	27.4%	<0.001
Previous MI	46	4.1%	9	2.2%	25	4.6%	12	6.8%	0.080
Previous CABG	41	3.6%	1	0.2%	9	1.7%	31	1.7%	<0,001
Previous stroke/TIA	117	10.4%	31	7.6%	61	11.2%	25	14.3%	0.037
COPD (FEV1<60%)	221	19.6%	50	12.3%	125	22.9%	46	26.3%	<0.001
NYHA III-IV class	599	53.2%	220	54.2%	261	4.9%	118	67.4%	<0.001
EuroSCORE II (median, 25th-75th iQR)	5.4	(3.2 - 7.3)	2.3	(1.5-2.9)	5,75	(5.6-7.2)	11.5	(8.0-12.0)	<0.001
eGFR <30 (mL/min/1,73 m2)	100	8.8%	10	2.5%	52	9.5%	38	21.7%	<0.001
LVEF (mean, SD)	57.9	11.0	60. 5	8.7	56.1	11.1	55.7	14.9	<0.001
LVEF<30	69	6.1%	6	1.5%	38	7.0%	25	14.3%	0.001
Preoperative MR>2	105	9.3%	25	6.2%	56	10.3%	24	13.7%	0.001
Bicuspid valve (Sievers 1)	68	6,.0%	35	8.6%	27	4.9%	6	3.4%	0.012

BSA: Body Surface Area; BMI: Body Mass Index; MI: Myocardial Infarction; CABG: Coronary Artery Bypass Grafting; BSA: Body Mass Index; TIA: Transitory Ischemic Attack; MR: mitral regurgitation; LVEF: Left Ventricular Ejection Fraction; COPD: Chronic Obstructive Pulmonary Disease; NYHA: New York Heart Association; FEV: Force Expiratory Volume; GFR: Glomerular Filtration Rate
